# Compromised Hindgut Microbial Digestion, Rather Than Chemical Digestion in the Foregut, Leads to Decreased Nutrient Digestibility in Pigs Fed Low-Protein Diets

**DOI:** 10.3390/nu14142793

**Published:** 2022-07-07

**Authors:** Junyan Zhou, Yuming Wang, Lu Wang, Jiayu Tu, Lijie Yang, Guangxin Yang, Xiangfang Zeng, Shiyan Qiao

**Affiliations:** 1 State Key Laboratory of Animal Nutrition, College of Animal Science and Technology, China Agricultural University, Beijing 100193, China; zjycau@163.com (J.Z.); wangyuming@caas.cn (Y.W.); wanglucau@163.com (L.W.); tujiayu0302@163.com (J.T.); yang.superman@163.com (L.Y.); gx_yang@126.com (G.Y.); ziyangzxf@163.com (X.Z.); 2 Beijing Bio-Feed Additives Key Laboratory, Beijing 100193, China; 3 The State Key Laboratory of Animal Nutrition, Institute of Animal Sciences, Chinese Academy of Agricultural Sciences, Beijing 100193, China

**Keywords:** low-protein diet, nutrient digestibility, in vitro fermentation, flora, growing pigs

## Abstract

Background: Reduced nutrient digestibility due to low-protein (LP) diets occurring in the foregut or hindgut of pigs remains unclear. Methods: Growing barrows (21.7 ± 1.7 kg) were allotted into LP and high-protein (HP) diet treatments. Ileal digesta and feces were collected for in vitro cross-fermentation and microbial sequencing, and cross-feeding assessed nutrient digestibility. Results: No difference in foregut digesta flora and nutrient digestibility between treatments was observed. LP diet caused decreased total tract digestibility of dry matter (DM), organic matter (OM), gross energy (GE), neutral detergent fiber (NDF), and acid detergent fiber (ADF) compared with the HP diet (*p* < 0.05). The fermentation broth from LP diet-fed pigs induced less full fermentation digestion of DM, OM, crude protein, and GE than HP broth (*p* < 0.05). Additionally, LP broth fermentation presented lower fermentation gas and short-chain fatty acids (SCFAs) generation than HP group (*p* < 0.05). This situation above may be related to decreased abundances of Lachnospiraceae, *Eubacterium_eligens_group*, *Roseburia*, and *Ruminococcaceae_UCG-009*, which can efficiently ferment nutrients to produce SCFA. Conclusions: Change in the flora caused compromise in hindgut microbial fermentation digestion leads to decreased total tract nutrient digestibility in pigs fed an LP diet.

## 1. Introduction

Adequate digestion is the foundation for the efficient utilization of nutrients. The digestion of dietary nutrients in the gastrointestinal tract of monogastric animals can be divided into two stages: chemical enzymatic digestion, which occurs mainly within the stomach, and small intestine and microbial digestion, which occurs mainly within the large intestine [1]. Nutrient digestion efficiency is affected by factors such as the type and quantity of the ingested nutrients and the region where digestion occurs [2].

Previous studies have demonstrated that under low-protein (LP) diet conditions, the growth performance of pigs and the nutrient digestibility of pigs are considerably reduced even when the limiting amino acid requirement is satisfied by industrial crystalline amino acid supplementation [3,4]. Researchers have suggested that this phenomenon may occur because a low dietary crude protein (CP) content lessens the stimulation of digestive enzyme secretion and thus weakens chemical digestion [5]. Others have deemed this to be because a low dietary CP content decreases the amount of nitrogen entering the hindgut and thus hinders the growth and proliferation of the hindgut microbes, which generally weakens the fermentation capacity of the microbiota [6].

Identifying the cause of the differences in nutrient digestibility between high-protein (HP) and LP diet feeding is of great significance for targeted optimization of nutrient efficiency and improved pig production performance. Therefore, pigs fed an HP or LP diet with a T-cannula at the distal ileum were used as experimental animals to collect the ileal digesta and feces for ileal and total tract nutrient digestibility determination. In addition, studies have indicated that there is a time delay between dietary regulation and changes in the gut microbiota [7,8]. Thus, the present experiment assumed that changes in the microbial fermentation performance in the days following pig diet changes can be ignored. Therefore, cross-feeding can evaluate the fermentability of the ileal digesta and the fermentation capacity of the fecal microbiota.

In vitro fermentation, gas production technology is often used to simulate the fermentation state of ruminant microorganisms [9], and in recent years, it has also been regarded as an appropriate approach to evaluate the fermentation characteristics of hindgut microorganisms in monogastric animals [10]. The gas production curve and fermentation digestibility of a substrate are important reflections of the rate and extent of microbial fermentation. Therefore, the current study analyzed the fecal microflora of pigs fed different diets and used their feces to manufacture bacterial broth. This broth was used to cross-ferment substrates made from the ileal digesta of pigs fed different diets for evaluation of the effect of dietary protein content on hindgut microbial fermentation.

## 2. Materials and Methods

Animal experiments were approved by the China Agricultural University Animal Care and Use Committee (Beijing, China, AW11102202-1-1). All experimental supplies and pigs were offered by the FengNing Swine Research Unit of China Agricultural University (Chengdejiuyun Agricultural and Livestock Co., Ltd., Hebei, China).

### 2.1. Experimental Diets and Pigs

**Figure 1:**
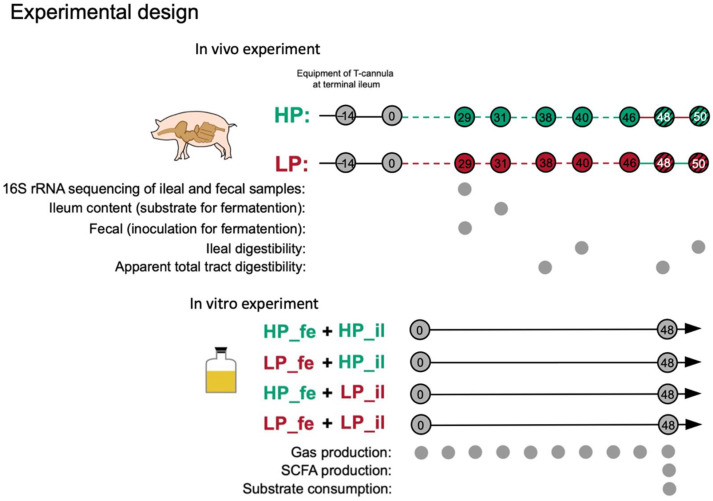
Experimental design. In vivo experiment: black line, adaptation, and recovery; green dotted line, normal high-protein diet feeding; red dotted line, normal low-protein diet feeding; red line, low-protein diet cross-feeding; green line, high-protein diet cross-feeding. Each sampling period lasted two days. In vitro fermentation: black line, continuous fermentation, and dense sampling for 48 h. Abbreviations: HP, high-protein diet treatment; LP, low-protein diet treatment; HP_fe + HP_il, high-protein diet treatment feces and high-protein diet treatment ileal digesta; LP_fe + HP_il, low-protein diet treatment feces and high-protein diet treatment ileal digesta; HP_fe + LP_il, high-protein diet treatment feces and low-protein diet treatment ileal digesta; LP_fe + LP_il, low-protein diet treatment feces and low-protein diet treatment ileal digesta.

Twelve crossbred (Duroc × Landrace × Yorkshire) barrows (21.7 ± 1.7 kg) fitted with a T-cannula at the terminal ileum were randomly allotted to receive 1 of 2 experimental diets, which resulted in 6 observations per dietary treatment. Dietary treatments were an HP diet and an LP diet. Experimental diets were formulated to provide sufficient or excess vitamins and minerals to the experimental animals according to the National Research Council [11] (Table 1). Experimental design was briefly described in Figure 1.

Pigs were housed individually in stainless steel metabolism crates (1.4 × 0.7 × 0.6 m). The daily feed intake of each pig was determined to be 2.8 times greater than the maintenance energy requirements (197 kcal per kg body weight^0.6^) [11]. The daily feed supplied to each pig was divided equally into two meals, given at 09:00 and 16:00 during the experimental period. The temperature was maintained at 23 ± 2 °C.

### 2.2. Experimental Design and Sample Collection for the In Vivo Trial

The experiment lasted for 51 d. Pigs were fed experimental diets for 28 days to stabilize the flora. On days 29 and days 30 of the experiment, pig feces were collected using sterile sampling bags and transferred immediately after adding glycerol and sterile saline in the proper proportions (feces: glycerol: saline = 3: 2: 15, *m*/*v*/*v*). Thereafter, each sterile bag was transferred to the refrigerator for storage. On days 31 and 32 of the experiment, ileal digesta samples were collected into plastic bags for fermentation substrate manufacture (Table 2). From days 38 to 49 and 40 to 41, fresh feces and ileal digesta samples were collected for digestibility determination, respectively. Plastic bags with 5 g of chlortetracycline were attached to the cannula barrel using an elastic plastic rope. Bags were removed at least once every 30 min and immediately stored at −20 °C. Low-temperature storage and antibiotic supplementation were used to weaken the microbial degradation of the nutrients in the digesta. From 16:00 on day 46, pigs were fed the opposite diets (HP-treated pigs were fed LP diets, and LP-treated pigs were fed HP diets). From days 48 to 49 and days 50 to 51, fecal and ileal digesta samples were collected for digestibility determination, respectively.

### 2.3. In Vitro Fermentation Assay

The in vitro fermentation assessment used 2 × 2 cross-fermentation. The buffers for the in vitro fermentation experiments were prepared based on previous studies [12]. Frozen digesta samples were thawed at room temperature. Ileal digesta from six pigs fed the same diet was thoroughly mixed and freeze-dried as fermentation substrate. Ileal digesta powder (500 mg), base solution (76 mL), vitamin/phosphate buffer (1 mL), carbonate solution (4 mL), and reducing solution (1 mL) were placed into a 100 mL anaerobic fermentation flask. Then, the flask was placed in a sealed sterile operation box continuously vented with nitrogen.

The process of bacterial broth inoculation was performed according to a previous study [13]. In brief, the fecal bacteria inoculum from each pig was thawed in the operation box, mixed evenly, filtered through four layers of sterile gauze, and accurately transferred to a fermentation bottle with 20 mL of the filtrate. In addition, fermentation broth from 6 pigs in the same treatment was mixed in the same proportion every three, and the mixed 2 bacterial liquids were transferred to a fermentation bottle as above. Therefore, in each treatment, 8 different fermentation broths (6 individual and 2 mixed) fermented the same fermentation substrate as 8 replicates. Afterward, the bottles were purged with anaerobic N_2_ for 5 s, sealed with a butyl rubber stopper and Hungate screw caps, and individually connected to the gas inlets of the 64-channel AGRS-III type microbial anaerobic fermentation microgas automatic recorder with medical plastic infusion pipes to continuously record the cumulative gas production. All bottles were incubated at 39 °C for 48 h. After fermentation, the fermentation broth was vacuum filtered through filter paper (Whatman 1541, pore size 22 μm) that had been previously dried and weighed in advance, and the filtered residue along with the filter paper was dried in an oven at 105 °C to a constant weight.

### 2.4. Chemical Analyses

The analyses of dry matter (DM), CP, and ash concentrations in the feed, digesta, and fermentation residue samples were conducted according to the Association of Official Analytical Chemists procedures [14]. The determination of neutral detergent fiber (NDF) and acid detergent fiber (ADF) was performed according to the method of van Soest et al. [15] using a filter bag and fiber analyzer (Ankom, NY, USA). The gross energy (GE) was measured using an automatic oxygen bomb calorimeter (Model 6400, Parr, Moline, IL, USA).

### 2.5. Short-Chain Fatty Acid Analyses

The concentration of short-chain fatty acids (SCFAs) in the fermentation supernatants was determined by a previous method [16]. The sum of the formate, acetate, propionate, butyrate, and isobutyrate contents was taken as the total SCFA, and valerate and isovalerate were not considered in this research.

### 2.6. Bacterial Community Structure

Bacterial DNA extraction was conducted using a QIAamp Fast DNA Feces Mini Kit (Qiagen Ltd., Düsseldorf, Germany). Bacterial 16S rRNA V3–V4 hypervariable region gene amplification was performed using a thermocycler polymerase chain reaction system (GeneAmp 9700, ABI, Carlsbad, CA, USA). The Illumina HiSeq 2500 platform (San Diego, CA, USA) was used to purify, quantify, pool, and sequence the resulting amplicons. The definition and removal of the nonnormal gene sequences were conducted by UCHIME.

### 2.7. Calculations

#### 2.7.1. Nutrient Digestibility

Apparent ileal digestibility (AID), apparent total tract digestibility (ATTD), and in vitro digestibility (%) = 100 − [(CCr input × CN output)/(CCr output × CN input) × 100].

In this equation, CCr input is the concentration of the index compound (Cr) in feed or pre-fermentation substrate, and CCr output is the concentration of the index compound (Cr) in the ileal digesta, feces, or fermentation residue; CN input is the concentration of a nutrient in the feed or pre-fermentation substrate, and CN output is the concentration of the nutrient in the ileal digesta, feces, or fermentation residue.

#### 2.7.2. Gas Production Profiles

The cumulative gas production at time t (GPt, mL/g DM) for each fermentation bottle was fitted to an exponential model (France, Dijkstra, Dhanoa, Lopez, and Bannink, 2000) using the nonlinear procedure of the software package SAS for Windows:

GP_t_ = A × (1 − e ^−C×t^)

where GP_t_ represents the cumulative gas production (GP) at time t; A represents the asymptotic gas production generated at a constant fractional rate (C) per unit time; e is the base of a natural logarithm; and t is the gas production time. The GP of 8 replicates at the same time point for each treatment were averaged and connected these averages data with a straight line.

AGPR = A × C/(2 × Ln2)

where AGPR represents the average gas production rate, which was defined as the average gas production rate between the start of the incubation and the time at which the cumulative gas production was half that of its asymptotic value.

### 2.8. Statistical Analysis

The PROC MIXED procedures of SAS version 9.4 (SAS Institute, Cary, NC, USA) were used to perform data analysis. All data were checked for normal distribution and homogeneous variance using the UNIVARIATE procedure. Data were analyzed using the ANOVA of SAS. Data obtained by ANOVA are shown as the means ± SD. Differences at a *p*-value ≤ 0.01 were considered highly significant, and differences at a *p*-value ≤ 0.05 were considered significant.

The α diversity of the fecal bacterial community was analyzed using the Mann–Whitney U test and Kruskal–Wallis test. The statistical significance of the principal coordinate analysis (PCoA) of microbial compositions between the treatments was performed using the QIIME software package (version 2) and was based on Bray–Curtis distance metrics. Linear discriminant analysis effect size (LEfSe) was used to compare differences in taxonomic levels, including phylum, class, order, family, and genus.

## 3. Results

### 3.1. In Vivo Digestibility

**Figure 2:**
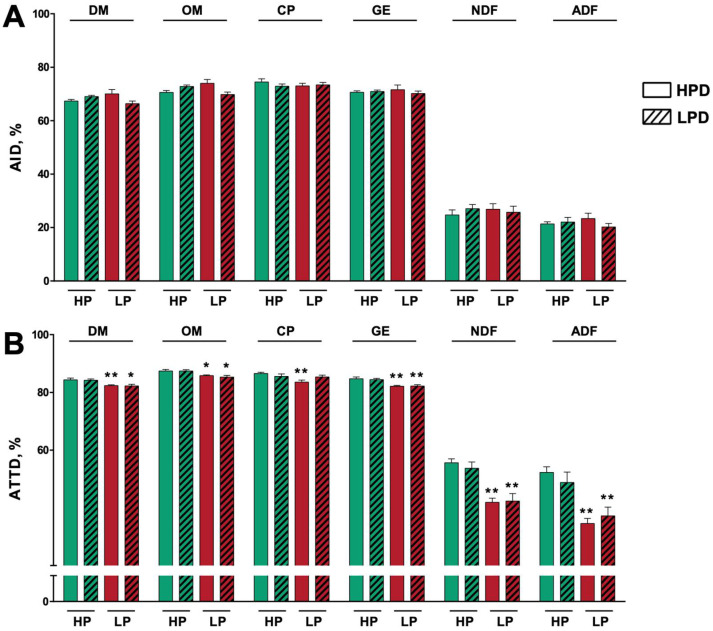
In vivo nutrient digestibility. Apparent ileal digestibility (**A**) and apparent total tract digestibility (**B**) of nutrients. Values are the means of 6 observations per treatment. * Significant difference compared with HP + HPD treatment (*p* ≤ 0.05); ** significant difference compared with HP + HPD treatment (*p* ≤ 0.01). Values are the least squares means ± SD; *n* = 6. Green bar, high-protein diet treatment; red bar, low-protein diet treatment. Abbreviations: HP, high-protein diet treatment; LP, low-protein diet treatment; HPD, high-protein diet feeding; LPD, low-protein diet feeding; AID, apparent ileal digestibility; ATTD, apparent total tract digestibility; DM, dry matter; OM, organic matter; CP, crude protein; GE, gross energy; NDF, neutral detergent fiber; ADF, acid detergent fiber.

There was no significant difference in the AID of the nutrients among treatments (Figure 2). The ATTD of GE, NDF, and ADF in the low-protein diet-treated pigs + high-protein diet feeding (LP + HPD) and the low-protein diet-treated pigs + low-protein diet feeding (LP + LPD) treatment groups were all lower than that in the high-protein diet-treated pigs + high-protein diet feeding (HP + HPD) treatment group (*p* < 0.01). Additionally, the ATTD of organic matter (OM) in the LP + HPD and LP + LPD treatment groups was modestly lower than that in the HP + HPD group (*p* < 0.05). Compared with the ATTD of DM in the pigs administered HP + HPD diet, that in the LP + HPD treatment group decreased significantly (*p* < 0.01) and that in the LP + LPD treatment group also decreased (*p* < 0.05). Regarding CP, the ATTD in the LP + HPD group was lower than that in the HP + HPD group (*p* < 0.01).

### 3.2. In vitro Fermentation Digestibility

**Figure 3:**
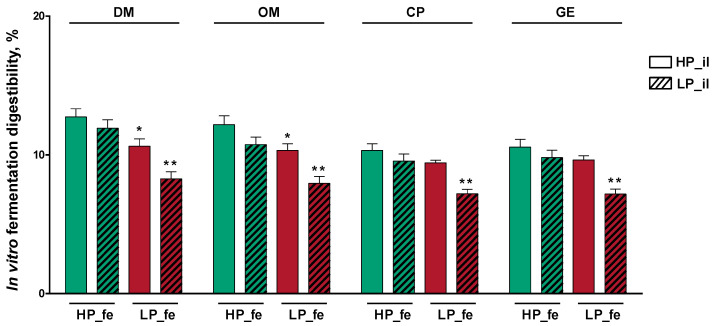
In vitro fermentation digestibility of nutrients. Values are the means of 8 observations per treatment. * Significant difference compared with HP_fe + HP_il treatment (*p* ≤ 0.05); ** Significant difference compared with HP_fe + HP_il treatment (*p* ≤ 0.01). Values are the least-squares means ± SD; *n* = 8. Green bar, high-protein diet treatment; red bar, low-protein diet treatment. Abbreviations: HP_il, high-protein diet treatment ileal digesta; LP_il, low-protein diet treatment ileal digesta; HP_fe, high-protein diet treatment feces; LP_fe, low-protein diet treatment feces; DM, dry matter; OM, organic matter; CP, crude protein; GE, gross energy.

The in vitro fermentation digestibility of DM, OM, CP, and GE in the LP_fe + LP_il group was significantly lower than those in the HP_fe + HP_il group (*p* < 0.01; Figure 3). Compared with the in vitro fermentation digestibility of DM and OM in the HP_fe + HP_il treatment, these values in the LP_fe + HP_il treatment group decreased moderately (*p* < 0.05).

### 3.3. Gas Production

**Figure 4:**
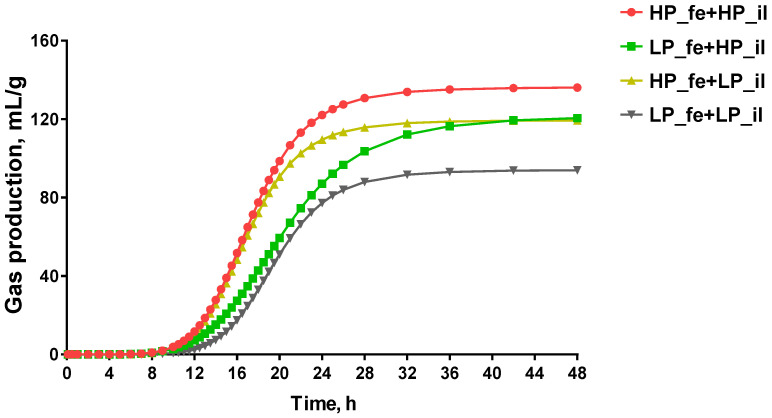
Cumulative gas production profiles from in vitro fermentation. Values are means, *n* = 8. Abbreviations: HP_fe + HP_il, high-protein diet treatment feces and high-protein diet treatment ileal digesta; LP_fe + HP_il, low-protein diet treatment feces and high-protein diet treatment ileal digesta; HP_fe + LP_il, high-protein diet treatment feces and low-protein diet treatment ileal digesta; LP_fe + LP_il, low-protein diet treatment feces and low-protein diet treatment ileal digesta.

**Figure 5:**
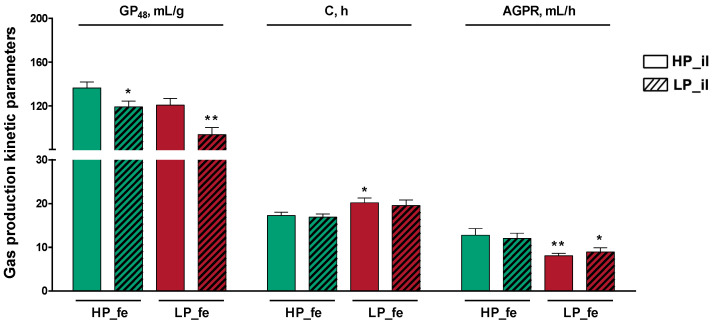
In vitro gas production kinetics and fermentation characteristics in the culture fluids after 48 h of incubation. Values are the means of 8 observations per treatment. * Significant difference compared with HP_fe + HP_il treatment (*p* ≤ 0.05); ** Significant difference compared with HP_fe + HP_il treatment (*p* ≤ 0.01). Values are the least-squares means ± SD; *n* = 8. Green bar, high-protein diet treatment; red bar, low-protein diet treatment. Abbreviations: HP_il, high-protein diet treatment ileal digesta; LP_il, low-protein diet treatment ileal digesta; HP_fe, high-protein diet treatment feces; LP_fe, low-protein diet treatment feces; GP_48_, gas production in 48 h; C, half-time of asymptotic gas production; AGPR, average gas production rate between the start of the incubation and the time at which the cumulative gas production was half that of its asymptomatic value.

The gas production curve is shown in Figure 4 and the fermentation kinetics parameters are presented in Figure 5. The high-protein diet treatment feces + high-protein diet treatment ileal digesta (HP_fe + HP_il) group showed the highest gas production at 48 h (GP_48_), which was higher than that in the low-protein diet treatment feces + low-protein diet treatment ileal digesta (LP_fe + LP_il) group (*p* < 0.01) and modestly higher than that in the low-protein diet treatment feces + high-protein diet treatment ileal digesta (LP_fe + HP_il) group (*p* < 0.05). Compared with the time at which the gas production volume reached 1/2 of the maximum gas production volume (C) in the LP_fe + HP_il treatment group, this value in the HP_fe + HP_il treatment group showed a slight increase (*p* < 0.05). Compared with the HP_fe + HP_il group, the AGPR in the LP_fe + HP_il and LP_fe + LP_il group decreased modestly (*p* < 0.01, *p* < 0.05, respectively).

### 3.4. SCFA Concentrations in the Fermentation Supernatants

**Figure 6:**
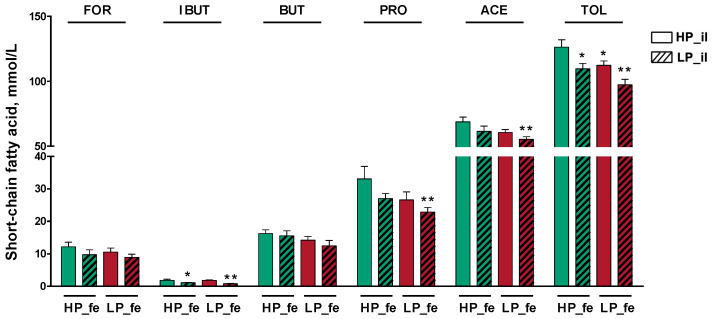
Quantification of the short-chain fatty acids in the fermentation supernatants. Values are the means of 8 observations per treatment. * Significant difference compared with HP_fe + HP_il treatment (*p* ≤ 0.05), ** Significant difference compared with HP_fe + HP_il treatment (*p* ≤ 0.01). Values are the least-squares means ± SD; *n* = 8. Green bar, high-protein diet treatment; red bar, low-protein diet treatment. Abbreviations: HP_il, high-protein diet treatment ileal digesta; LP_il, low-protein diet treatment ileal digesta; HP_fe, high-protein diet treatment feces; LP_fe, low-protein diet treatment feces; FOR, formate; IBUT, isobutyrate; BUT, butyrate; PRO, propionate; ACE, acetate; TOL, total short-chain fatty acids.

The isobutyrate concentrations in the high-protein diet treatment feces + low-protein diet treatment ileal digesta group and LP_fe + LP_il groups was lower than that in the HP_fe + HP_il group (*p* < 0.05, *p* < 0.01, respectively; Figure 6). LP_fe + LP_il treatment produced decreases in acetate and propionate concentrations compared with pigs undergoing HP_fe + HP_il treatment (*p* < 0.01). Compared with the HP_fe + HP_il treatment group, the HP_fe + LP_il, LP_fe + HP_il, and LP_fe + LP_il treatment groups both presented lower total SCFA concentrations (*p* < 0.05, *p* < 0.05, *p* < 0.01, respectively).

### 3.5. Bacterial Community

**Figure 7:**
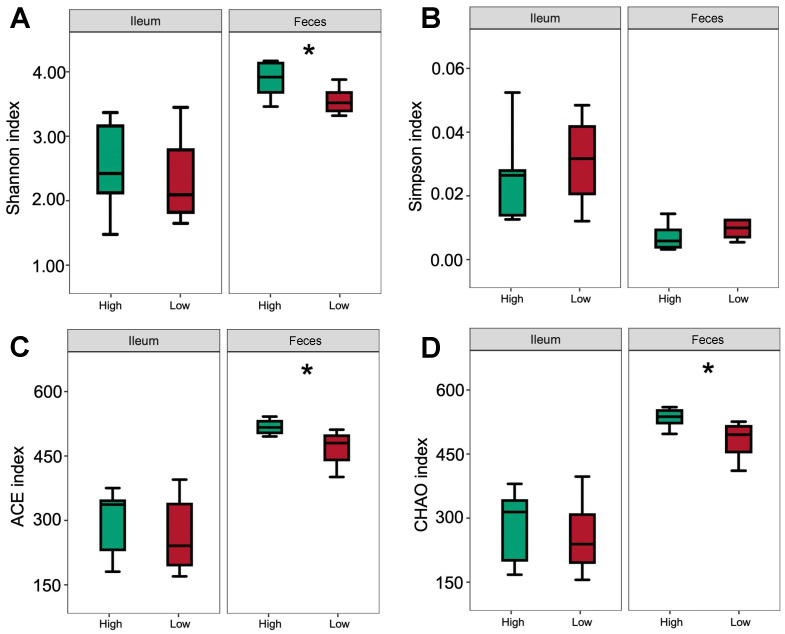
The α diversity of the fecal bacterial community. Shannon index (**A**), Simpson index (**B**), ACE index (**C**), and CHAO index (**D**) of the fecal bacterial community. * Significant differences between treatments (*p* ≤ 0.05). Green box, high-protein diet treatment; red box, low-protein diet treatment.

**Figure 8:**
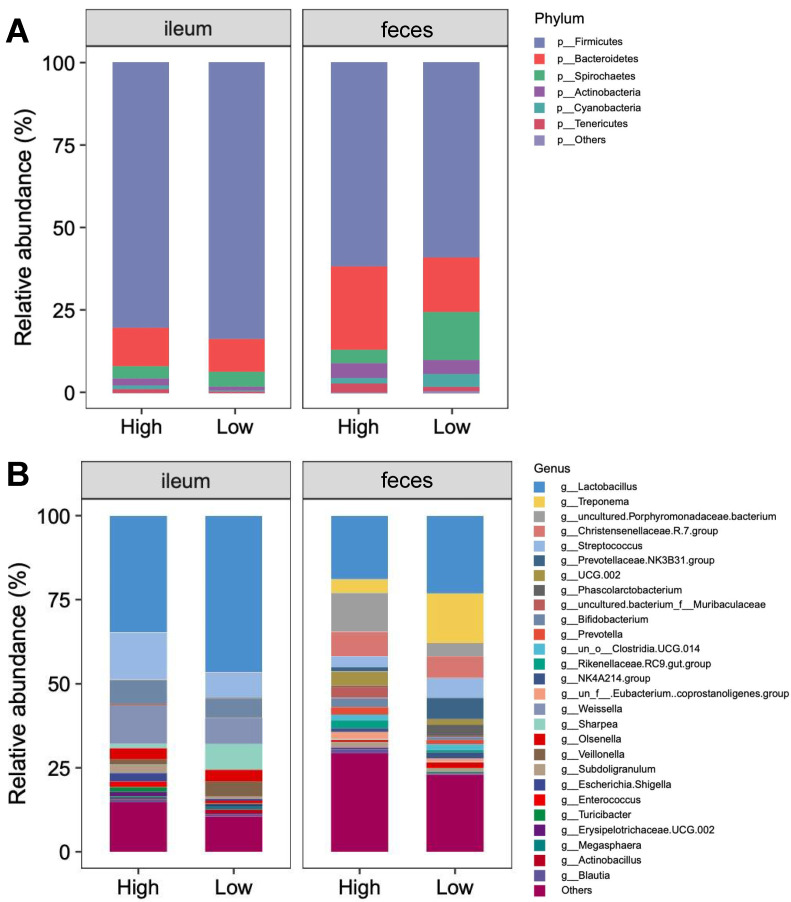
Fecal bacterial community at the phylum and genus levels in the ileal digesta and feces. Microbial community bar plots of the phyla with an abundance of 0.015% or greater (**A**) and the families with a proportion of 0.015% or higher (**B**).

There were no significant differences in the diversity and richness of the bacterial community in the ileal digesta among the pigs receiving different treatment diets (Figure 7). Phylum-level analysis proved that the microbiota composition in the pig ileal digesta of pigs was consistently dominated by Firmicutes (82.00%; Figure 8), Actinobacteriota (10.71%), and Proteobacteria (4.09%). At the genus level, *Lactobacillus* (40.13%), *Streptococcus* (10.61%), and *Weissella* (9.18%) were the dominant bacteria. 

The Shannon, ACE, and CHAO indices in the feces of the pigs after HP treatment were higher than those in the feces of pigs receiving LP treatment (*p* < 0.05; Figure 7). To further determine changes in the gut microflora, the bacterial communities were analyzed at the phylum and genus levels (Figure 8). The results showed that at the phylum level, Firmicutes (60.40%), Bacteroidetes (20.74%), and Spirochaetes (9.28%) were the dominant bacteria among all groups; at the genus level, *Lactobacillus* (20.53%) and *Treponema* (9.10%) were the dominant bacteria.

**Figure 9:**
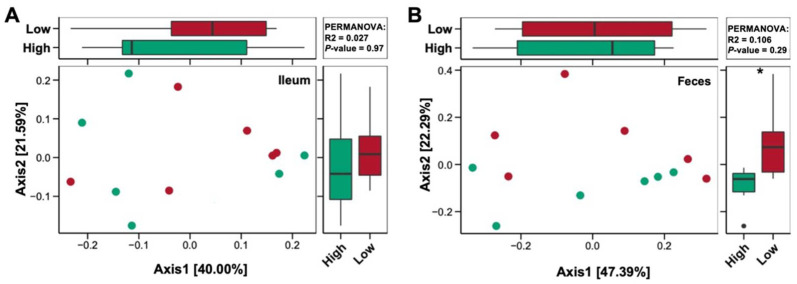
Principal coordinate analysis. Principal coordinate analysis of the microbiota from the ileal digesta (**A**) and feces (**B**) of pigs fed a high-protein diet (green points and green boxes) or a low-protein diet (red points and red boxes); *n* = 6. The distances between the symbols on the ordination plot reflect the relative dissimilarities in the community structures.

The assessment of β-diversity differences based on the OTUs in the ileal digesta and fecal microbiota is illustrated in Figure 9. The PERMANOVAs of the unweighted UniFrac distances revealed distinct clustering patterns in the ileal digesta (*p* = 0.97) and fecal (*p* = 0.29) microbiota between the HP and LP treatments. It is worth noting that the PERMANOVAs of the feces showed that the microbial communities after HP and LP treatments differed significantly on Axis 2 (22.29%; *p* < 0.05).

**Figure 10:**
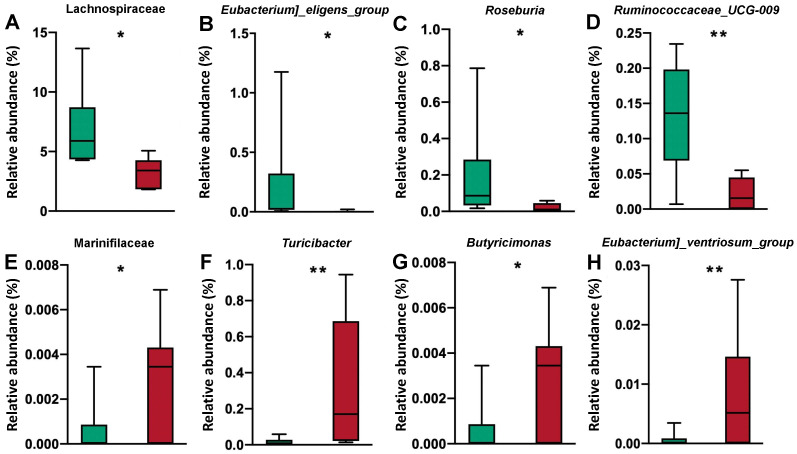
Analysis of all differential bacteria in the feces from phylum to genus. Relative abundance of bacteria (**A**–**H**) in the feces of pigs fed a high-protein diet (green boxes) or a low-protein diet (red boxes); *n* = 6. * Significant difference compared with HP_fe + HP_il treatment (*p* ≤ 0.05), ** Significant difference compared with HP_fe + HP_il treatment (*p* ≤ 0.01).

Significant differences in the microbial community among the different treatment groups are shown in Figure 10. There were no differences in the abundance of any microorganism from the ileal digesta between the two treatment groups. In feces, many bacteria, such as Lachnospiraceae, *Eubacterium_eligens_group*, *Roseburia*, and *Ruminococcaceae*_*UCG*-*009*, were more abundant in the HP treatment group than in the LP treatment group; additionally, other bacteria, such as *Eubacterium_ventriosum_group*, *Turicibacter*, Marinifilaceae, and *Butyricimonas*, were less abundant after HP treatment than after LP treatment.

## 4. Discussion

By supplementing crystalline amino acids, LP diets can precisely satisfy the amino acid requirements of livestock and poultry while saving feed costs and reducing nitrogen pollution via excretion, which is considered the key to efficient animal husbandry [17]. However, recent studies have demonstrated that even when the limiting amino acid nutrient requirements are satisfied, the growth performance of pigs fed LP diets is impaired, which may be related to the compromise in nutrient digestibility [3,4,18]. In the present experiment, cross-feeding, microbial flora analysis, and in vitro fermentation assays were applied to investigate the focal point of the effects of dietary CP content on nutrient digestibility in pigs. The results showed that decreased dietary CP content had no clear influence on the microflora structure and nutrient digestibility in the pig foregut but decreased the hindgut richness and diversity of the microflora and the abundances of *Roseburia*, Lachnospiraceae, *Ruminococcaceae*_*UCG*-*009*, and *Eubacterium_eligens_group*, which subsequently undermine the fermentation capacity of the hindgut microbial flora and eventually the total tract nutrient digestibility.

All vertebrates need to achieve the same goal, which is to convert macromolecular nutrients into constituent molecules (i.e., free fatty acids, monosaccharides, amino acids, etc.) that can be absorbed to be used as structural molecules and energy substrates. Conversion efficiency is an important consideration for agriculture, in which animal feed is an input cost. Digestion is the most basic and critical link in the conversion of nutrients. Digestion of ingested nutrients includes chemical enzymatic digestion, which mainly occurs in the stomach and small intestine, and microbial digestion, which mainly occurs in the large intestine. As early as 1964, Snook found that dietary protein can stimulate the synthesis and secretion of various digestive enzymes and also delay the degradation of these enzymes in the intestine to achieve efficient nutrient digestion [19]. Corring suggested that any alterations in the type or quantity of dietary proteins can lead to an adjustment in specific and total enzymatic activities in the pancreatic tissue and the pancreatic juice, and the brush border enzyme activities of rats eating the same amount of food increased with a protein-rich diet [20]. In the current experiment, the AID of nutrients in pigs did not change when the dietary CP content was reduced by 5%, which may be because the slight decrease in dietary CP content was not sufficient to induce changes in the chemical digestion capacity. Notably, Wang et al. found that reducing the dietary CP content by 6 percentage points did not alter jejunal disaccharidase, protease, or lipase activities in growing pigs [18].

The diets in the present study were powdered feed with a diameter of about 1.5–2 mm mainly composed of corn and soybean meal, which resulted in poor digestibility, allowing a great number of nutrients that were not fully digested in the foregut to enter the post-gut for fermentation. Few endogenous digestive enzymes are in the hindgut of monogastric animals, so nutrient digestion here mainly depends on the fermentation of microorganisms [21]. Nitrogen is an essential substrate for microbial growth. The effects of dietary protein content on ruminant gut microbial growth and fermentation capacity have been extensively studied [22,23]. Pathak pointed out that degradable protein is a key to rumen microbial growth, and microbial protein synthesis is dependent on suitable nitrogen and carbohydrate sources [24]. Broderick found that an appropriate increase in dietary CP content improved rumen microbial growth and milk production in dairy cows [25]. Other studies have investigated the effect of dietary protein on monogastric animal gut microbial composition and activity, and total protein intake was identified as a major factor that eventually affects the extent of protein fermentation in the intestines of rats. Additionally, weaned piglets fed an LP diet exhibited a reduction in protein fermentation activity [26]. In the current experiment, the AID of nutrients was similar among the different treatments. It was found that the LP diet reduced the ATTD of nutrients in pigs, while nutrient digestibility was improved after the HP diet-fed pigs were cross-fed with the LP diet. These data confirm the crucial influence of dietary protein content on the fermentation capacity of the animal gut microbiota and present the novel view that LP diets reduce hindgut microbial nutrient fermentation, which then reduces the ATTD of nutrients. This study mainly focused on the effect of dietary crude protein content on nutrient digestion and fermentation in actual pig production, so this goal was achieved by changing the dietary content of corn and soybean meal. Perhaps a homozygous or semi-homogenous diet could be used to make more detailed exploration in the future.

The in vitro fermentation digestibility of nutrients and the gas production kinetic characteristics reflect the fermentability of the substrate and the fermentation capacity of the fermentation broth [27]. The sampling points of the in vitro fermentation GP experiment are dense, and it is cumbersome and meaningless to compare each time point. Therefore, the differences in the GP of different treatment groups at the same time point were not investigated, and the comparison of 3 indicators that reflect GP characteristics between treatments was conducted. The GP_48_ values, which are the gas production values per gram of substrate in 48 h, of the LP_fe + LP_il and HP_fe + LP_il groups of pigs were lower than that of the HP_fe + HP_il pigs, thus representing poor fermentability of the ileal digesta and a weak fermentation capacity of the fermentation broth after LP treatment. C, which refers to the half-time of asymptotic gas production, was found to be higher in the LP_fe + HP_il group than in the HP_fe + HP_il group. This result may be because the fermentation capacity of LP_fe is worse than that of HP_fe, and compared with HP_fe, more time is required for LP_fe to ferment all of the fermentable, rich nutrients in HP_il. The decreased AGPR of the LP_fe + HP_il and LP_fe + LP_il treatments compared with HP_fe + HP_il treatment further confirmed that the LP diet diminished the fermentation ability of the microorganisms in the hindgut.

SCFAs are produced mainly through the saccharolytic fermentation of carbohydrates that escape digestion and absorption in the foregut [28]. SCFAs absorbed in the colon contribute 6–10% of the total energy requirements in humans, and their contribution likely increases in humans who ingest more dietary fiber [29,30]. It has been confirmed that in addition to an energy supply, SCFAs produced by fermentation of hindgut flora can contribute to energy metabolism modulation and health [31]. In this experiment, the feces from the pigs administered different treatments were used as a bacterial broth to cross-ferment digesta in vitro. In this way, the SCFA concentration in the fermentation supernatant can not only compare the effects of LP and HP diets on the production of SCFA in the hindgut of pigs but also evaluate the potential of the ileal digesta to generate SCFAs by fermentation and the capacity of hindgut microbes to ferment undigested nutrients to nourish the host. The experimental results proved that HP_fe fermented the substrate to generate more acetate, propionate, isobutyrate, and total SCFAs than those produced by LP_fe; in addition, HP_il was able to generate more acetate, propionate, and total SCFAs through fermentation than LP_il.

The isobutyrate data in the present experiment are consistent with previous studies showing that HP diets lead to the generation of more branched-chain fatty acids. As a branched-chain fatty acid, isobutyrate is produced by hindgut microorganisms’ fermentation of branched-chain amino acids, and high concentrations of fecal isobutyrate may be linked to impaired hindgut health [32]. Acetate is the principal SCFA absorbed into the blood from the hindgut and also an important energy source for tissues such as the liver, where acetate is used for lipogenesis and cholesterol synthesis [33]. Acetate is also utilized by muscles and other tissues, where it can be metabolized for energy [34]. In the current study, the strong fermentative acetogenic ability of HP_fe may be related to the flora. Compared with the LP diet group, the abundance of the Firmicutes species *Eubacterium eligens* in the feces of the HP group was significantly increased. *Eubacterium eligens* is an important, specialized degrader of diet-derived pectins in the colon that produces a constitutive pectate lyase, and acetate is the main product of the *Eubacterium eligens* fermentation of pectin [35]. In addition to *Eubacterium eligens*, the high concentration of acetate in the supernatant of the HP_fe group may also be related to the elevated abundances of Lachnospiraceae and *Ruminococcaceae*_*UCG*-*009* because Lachnospiraceae and Ruminococcaceae contain a high proportion of putative acetogens, which has been confirmed before [36]. Another interesting finding in our research was that the increase in the abundance of the well-known butyrate-producing bacteria *Roseburia* in the HP group did not result in an increase in the butyrate concentration in the HP_fe group. *Roseburia* was enriched in the HP group, possibly due to the higher acetate concentration in HP_fe. In addition to fiber, acetate can also be used as the main nutrient substrate for the growth and proliferation of *Roseburia*, and the rich contents of acetate in HP_fe nourish *Roseburia* [37]. Many studies have suggested that changes in the abundance of *Roseburia* are consistent with beneficial glucose metabolism. *Roseburia* is probiotic bacteria that maintain intestinal physiology and immune homeostasis and have a strong ability to degrade resistant starch or nonstarch polysaccharides to produce SCFAs [38,39].

Propionate is a known precursor for hepatic gluconeogenesis [40]; indeed, it has been estimated that approximately 90% of the propionate in the portal vein is extracted by the liver [41], and glucose synthesis from propionate accounts for 69% of total glucose production [42]. The elevated abundance of Lachnospiraceae and *Ruminococcaceae*_*UCG*-*009* may be the pivotal reason for the elevated supernatant concentration of propionate and improved nutrient fermentation digestibility of the HP_fe group. Studies have shown that some Lachnospiraceae and Ruminococcus species can synthesize propionate through the propylene glycol pathway using carbohydrates such as fucose or rhamnose as substrates [38]. Lachnospiraceae bacteria have been verified to generate cellulase, which plays a vital role in the decomposition of fiber in the gut [43]. Lachnospiraceae also have a considerable capacity to utilize diet-derived polysaccharides, including starch, inulin, and arabinoxylan [44]. A previous study suggested that a decrease in Lachnospiraceae abundance is likely to have negative health implications because this family of bacteria has numerous beneficial functions, such as converting primary bile acids to secondary bile acids [45] and producing an important class of peptide antibiotics [46]. The improved post-gut fermentative capacity in the HP_fe group may be related to the enhanced functions of Lachnospiraceae.

Ruminococcaceae has been associated with the utilization of resistant starch in ruminants and humans [47,48]. A previous study reported that beef cattle with high feed efficiency had a higher relative abundance of Ruminococcaceae in the gut [49]; higher weight gain and ruminal volatile fatty acid concentrations were found in cattle-yaks with a higher relative abundance of Ruminococcaceae [50]. The abundance of human fecal Ruminococcaceae after fecal microbial transplantation is positively correlated with SCFA concentration [51]. It has been proven that Ruminococcaceae are significantly positively correlated with fecal propionate and isobutyrate concentrations, which is consistent with the present study [52].

Many previous studies have reported that an appropriate reduction in dietary CP content can effectively improve the intestinal health of pigs and alleviate post-gut inflammation [17,53]. In the present study, the LP diet induced an increase in the abundance of fecal *Turicibacter*. Other studies showed that a reduction in *Turicibacter* contents can lead to ‘gut dysbiosis’, inducing a disruption of the epithelial barrier and ultimately resulting in increased serum IL-2 levels [54]. In addition, the levels of *Turicibacter* decreased in dog models of idiopathic inflammatory bowel diseases; as *Turicibacter* is anti-inflammatory, it has been shown to relieve kidney damage in mouse models [55]. *Butyricimonas*, which was enriched in the LP treatment group, is also thought to be positively correlated with health. For example, the high abundance of *Butyricimonas* in the rumen can produce SCFAs and then reduce the colonization of opportunistic pathogens in the intestines [56]; the contents of *Butyricimonas* decreases after porcine epidemic diarrhea virus infection, and a reduction in *Butyricimonas* has been noted in numerous autoimmune and inflammatory diseases [57]. Thus, while the LP group displayed weakened fermentation of nutrients in the hindgut, these animals may have demonstrated reduced inflammation and improved gut health.

## 5. Conclusions

LP diet administration decreased the abundances of *Eubacterium eligens*, Lachnospiraceae, *Ruminococcaceae*_*UCG*-*009*, and *Roseburia* in the hindgut of growing pigs, which weakened the fermentation capacity of microflora and thus impaired the nutrient digestion efficiency in the hindgut, and ultimately reduced the total tract nutrient digestibility in pigs fed an LP diet. These observations contribute to a better understanding of potential mechanisms that dietary CP content affects nutrient digestion and imply effective strategies for achieving enhanced nutrient utilization through optimizing microflora structure.

## Figures and Tables

**Table 1:** Ingredients and nutrient compositions of the experimental diets (%, as-fed basis).

Item	HP	LP
Ingredient, %		
Corn	58.60	71.70
Soybean meal	36.50	15.40
Wheat bran	1.50	8.90
Soybean oil	0.80	-
Limestone	0.86	0.92
Dicalcium phosphate	0.90	1.05
Salt	0.30	0.30
L-Lysine·HCl	0.03	0.59
L-Threonine	0.01	0.24
DL-Methionine	-	0.17
L-Tryptophan	-	0.09
L-Valine	-	0.14
Premix ^1^	0.50	0.50
Analyzed nutrient level		
Dry matter, %	90.20	88.97
Gross energy, MJ/kg	16.85	16.27
Crude protein, %	20.54	15.29
Ether extract, %	2.99	2.69
Ash, %	5.11	4.53
Acid detergent fiber, %	3.89	3.96
Neutral detergent fiber, %	11.35	12.03
Calculated nutrient level		
Net energy, MJ/kg	10.04	10.04
SID Lysine, %	1.00	1.00
SID Methionine + cysteine, %	0.59	0.59
SID Threonine, %	0.63	0.63
SID Tryptophan, %	0.21	0.21
SID Valine, %	0.79	0.66

^1^ Premix provided the following per kg of complete diet for growing pigs: vitamin A, 5512 IU; vitamin D3, 2200 IU; vitamin E, 64 IU; vitamin K3, 2.2 mg; vitamin B12, 27.6 μg; riboflavin, 5.5 mg; pantothenic acid, 13.8 mg; niacin, 30.3 mg; choline chloride, 551 mg; Mn, 40 mg (MnSO4); Fe, 100 mg (FeSO4∙H2O); Zn, 100 mg (ZnSO4); Cu, 100 mg (CuSO4∙5H2O); I, 0.3 mg (KI); Se, 0.3 mg (Na2SeO3). Abbreviations: HP, high-protein diet treatment; LP, low-protein diet treatment.

**Table 2:** Chemical compositions of the fermentation substrate digesta.

Diet	DM, %	OM, %	CP, %	GE, kcal/kg	NDF, %	ADF, %
HP	91.5	86.0	16.5	3651	26.5	9.5
LP	91.8	86.6	14.1	3772	30.5	10.6

Abbreviations: HP, high-protein diet treatment; LP, low-protein diet treatment; DM, dry matter; OM, organic matter; CP, crude protein; GE, gross energy; NDF, neutral detergent fiber; ADF, acid detergent fiber.

## Data Availability

The data supporting the reported results and conclusions can be found in the submitted figure and tables. Additional research materials and protocols that are relevant to the study are available from the corresponding author upon reasonable request.
